# Antimicrobial potential of known and novel probiotics on in vitro periodontitis biofilms

**DOI:** 10.1038/s41522-023-00370-y

**Published:** 2023-01-21

**Authors:** Wannes Van Holm, Rita Carvalho, Lize Delanghe, Tom Eilers, Naiera Zayed, Fabian Mermans, Kristel Bernaerts, Nico Boon, Ingmar Claes, Sarah Lebeer, Wim Teughels

**Affiliations:** 1 Department of Oral Health Sciences, University of Leuven (KU Leuven), Leuven, Belgium; 2 Centre for Microbial Ecology and Technology (CMET), Ghent University (UGent), Gent, Belgium; 3 Department of Bioscience Engineering, University of Antwerp, Antwerp, Belgium; 4 Faculty of Pharmacy, Menoufia University, Shibin el Kom, Egypt; 5 Bio- and Chemical Systems Technology, Reactor Engineering and Safety, Department of Chemical Engineering, University of Leuven (KU Leuven), Leuven, Belgium; 6 YUN NV, Niel, Belgium

**Keywords:** Plaque, Biofilms, Microbial ecology, Symbiosis, Microbiome

## Abstract

Several oral diseases are characterized by a shift within the oral microbiome towards a pathogenic, dysbiotic composition. Broad-spectrum antimicrobials are often part of patient care. However, because of the rising antibiotic resistance, alternatives are increasingly desirable. Alternatively, supplying beneficial species through probiotics is increasingly showing favorable results. Unfortunately, these probiotics are rarely evaluated comparatively. In this study, the in vitro effects of three known and three novel *Lactobacillus* strains, together with four novel *Streptococcus salivarius* strains were comparatively evaluated for antagonistic effects on proximal agar growth, antimicrobial properties of probiotic supernatant and the probiotic’s effects on in vitro periodontal biofilms. Strain-specific effects were observed as differences in efficacy between genera and differences within genera. While some of the *Lactobacillus* candidates were able to reduce the periodontal pathobiont *A. actinomycetemcomitans*, the *S. salivarius* strains were not. However, the *S. salivarius* strains were more effective against periodontal pathobionts *P. intermedia*, *P. gingivalis*, and *F. nucleatum*. Vexingly, most of the *Lactobacillus* strains also negatively affected the prevalence of commensal species within the biofilms, while this was lower for *S. salivarius* strains. Both within lactobacilli and streptococci, some strains showed significantly more inhibition of the pathobionts, indicating the importance of proper strain selection. Additionally, some species showed reductions in non-target species, which can result in unexpected and unexplored effects on the whole microbiome.

## Introduction/state-of-the-art

Oral pathologies, such as periodontitis, peri-implantitis, halitosis, and tooth decay are characterized by dysbiosis, a state of imbalance between the host and its microbiome. Prevention and treatment of these diseases focus on the suppression of the microbiome through mechanical removal often combined with the use of antibiotics and antiseptics. While this might initially prevent or resolve the pathology, it is questionable if these treatments can maintain or restore the microbiome in a commensal symbiotic state. Although decreases in the total subgingival microbiota of up to 3-log values can easily be achieved, a recolonization primarily by less putative oral pathogens towards baseline numbers occurs within one to two weeks^1^. This implies a need for continuous re-treatment of periodontitis patients. Periodontitis forms a lifelong threat for the patients, thus creating serious health and socio-economic burdens. The added value of the adjunctive use of antiseptics and antibiotics in the treatment of periodontitis remains controversial although clinical benefits have been shown^2^. The increasing prevalence of antibiotic-resistant bacteria favours the development of approaches that do not rely on antibiotics. It has been suggested that oral bacteria are also becoming more tolerant for the most common and often daily used oral antiseptics^3^. Increased tolerance of oral pathogens has also been linked to increased antibiotic resistance^3^.

This has caused a growing interest in pro-microbial approaches to modulate the oral microbiome towards a commensal state. One such approach is the direct application of live microorganisms that can confer a health benefit to the host, commonly referred to as probiotics^4^. While several probiotic strains have shown beneficial effects for oral health^5–11^, other studies assert a more limited benefit for their use^12–15^. Though the current opinion on oral probiotics may seem mixed, differences in strain effectiveness are often not taken into consideration. Different probiotic strains are grouped likely due to the lack of sufficient studies on individual strains. Systematic reviews of gastrointestinal research suggest that the effectiveness of probiotic treatment is highly strain- and disease specific^16^. Poorly chosen probiotics are very unlikely to show beneficial effects.

In oral research, as long as the probiotic is considered safe, the preclinical evaluation is often forgone and due to limitations in sample size, usually, only a small collection of probiotics is immediately evaluated in a clinical trial. However, the quest to find novel and clinically effective probiotics is hampered by the agar-based screening techniques. Despite being accessible and reliable, these are usually time and labour intensive with results that are only indicative of potential probiotic effects. Another limitation of current oral probiotic research is the evaluation of the probiotic’s incorporation within the oral ecology and its effects on not only pathobionts but also on commensal species, which are essential in maintaining oral health^17^.

In the current study, three known and seven novel probiotic candidates were selected from a collection of unique strains isolated from different sources: human niches and fermentation sources. In this study, the selected strains were comparatively evaluated in vitro on their inhibitory effects on periodontal pathobionts with proximal agar growth, antimicrobial properties of probiotic supernatant, and the probiotic’s incorporation in periodontal biofilms and the resulting changes in biofilm ecology.

## Results

### Agar inhibition assay of pathobionts

Using an agar-based inhibition assay, the influence of the probiotic candidates on the growth of the periodontal pathobionts was evaluated (Table 1).

**Table 1:** Inhibition of pathobiont growth on agar next to probiotic candidates.

	Inhibition ratio			
	*A. actinomycetemcomitans*	*P. intermedia*	*P. gingivalis*	*F. nucleatum*
*L. rhamnosus* GG	**0,352** ± **0,049**	0,034 ± 0,040	**0,672** ± **0,071**	**0,291** ± **0,077**
*L. pentosus* KCA1	**0,486** ± **0,081**	**0,319** ± **0,092**	**0,631** ± **0,058**	**0,244** ± **0,114**
*L. plantarum* WCFS1	**0,198** ± **0,110**	**0,368** ± **0,045**	**0,931** ± **0,089**	**0,401** ± **0,069**
*L. reuteri* V038	0,000 ± 0,000	0,000 ± 0,000	0,000 ± 0,000	0,000 ± 0,000
*L. fermentum* V001	0,000 ± 0,000	0,000 ± 0,000	0,000 ± 0,000	0,000 ± 0,000
*L. reuteri* F471	**0,337** ± **0,027**	0,000 ± 0,000	**0,716** ± **0,242**	0,000 ± 0,000
*S. salivarius* AMBR074	**0,413** ± **0,064**	**0,190** ± **0,043**	**0,716** ± **0,249**	**0,209** ± **0,080**
*S. salivarius* AMBR075	**0,415** ± **0,090**	**0,220** ± **0,063**	**0,750** ± **0,229**	**0,231** ± **0,032**
*S. salivarius* AMBR024	**0,410** ± **0,074**	**0,184** ± **0,066**	**0,674** ± **0,229**	**0,185** ± **0,069**
*S. salivarius* AMBR158	**0,467** ± **0,085**	0,000 ± 0,000	**0,822** ± **0,171**	**0,235** ± **0,056**

Data are expressed as the inhibition ratio (± standard deviation) between the edge of the probiotic’s spot and the pathobiont’s spot, relative to the diameter of the pathobiont’s control spot (Supplementary Fig. 1). Bold values represent statistically significant differences (ANOVA with Tukey HSD; *p* < 0.05) from the control spot (*n* = 9).

WCFS1 caused the strongest decreases of *P. intermedia*, *P. gingivalis* and *F. nucleatum*, but a weaker inhibition of *A. actinomycetemcomitans* than the other strains. LGG, KCA1, AMBR074, AMBR075, AMBR024 and AMBR158 had similar inhibitory effects on *A. actinomycetemcomitans*, *P. gingivalis* and *F. nucleatum*. Additionally, KCA1, AMBR074, AMBR075 and AMBR024 also showed significant antagonism towards *P. intermedia*, while LGG, F471 and AMBR158 did not. Finally, V038 and V001 did not display any inhibition towards the pathobionts.

### Flow cytometric (FCM) assay of probiotic cell-free supernatant inhibition of pathobionts

**Fig. 1:**
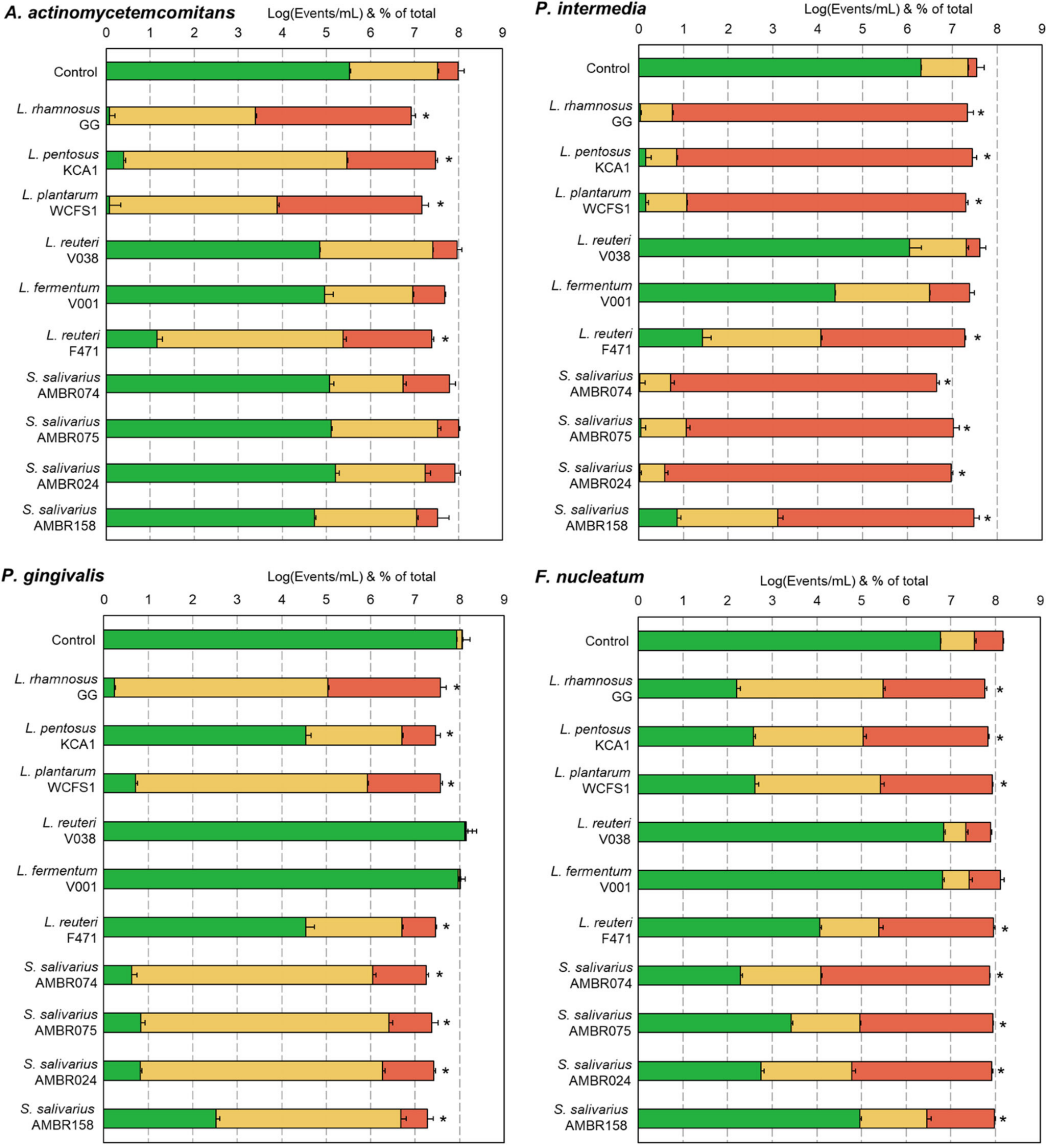
Antimicrobial properties of probiotic’s cell-free supernatant. Flow cytometric quantification of antimicrobial properties of the cell-free supernatant (CFS) of the probiotic candidates (postbiotic) on the membrane permeability, as a proxy for viability, of periodontal pathobionts after 6 h of incubation in the postbiotic. Data presented as mean and standard deviation of log_10_ of total events per mL (Log(Events/mL)) and percentage composition of the total divided into classes according to their permeability/viability: green = intact/live, red = permeable/dead and yellow = intermediate permeability/partially damaged membrane. Gates were defined according to the degree of red fluorescence through the entry of propidium iodide into healthy- and killed cultures (Supplementary Figs. 2 & 3). Statistical differences in remaining live cells compared to the control (*n* = 3, ANOVA with Tukey HSD) are depicted with an asterisk.

Using the double dye FCM assay, the probiotic’s cell-free supernatant (which can be considered as a postbiotic^18^) was evaluated if the secretome of the probiotic contains compounds able to affect the membrane permeability of the periodontal pathobionts. After defining the live- (intact, propidium iodide impermeable cells; low red fluorescence (PerCP-Cy5)), intermediate- and dead populations individually for *A. actinomycetemcomitans*, *P. intermedia*, *P. gingivalis* and *F. nucleatum* (Supplementary Figs. 2 & 3), the effect postbiotic CFS was observed after 6 h (Fig. 1).

In decreasing pathobiont killing, candidates LGG, WCFS1, KCA1 and F471 showed inhibition of *A. actinomycetemcomitans*. *P. intermedia* was inhibited in close to equal strength by LGG, WCFS1, KCA1, AMBR074, AMBR075, AMBR024 and followed in decreasing strength by AMBR158 and F471.

LGG showed the strongest inhibition of *P. gingivalis*, followed by a close to equal inhibition by WCFS1, AMBR074, AMBR075 and AMBR024. AMBR158, KCA1 and F471 displayed a lower inhibition of *P. gingivalis*.

For *F. nucleatum*, candidates LGG, WCFS1, KCA1, AMBR074, AMBR024 showed an equal inhibition, followed by a decreasing inhibition by AMBR075, F471 and AMBR158.

Candidates V038 and V001 displayed low to no antagonism toward the periodontal pathobionts.

Overall, LGG’s- and WCFS1’s CFS showed the strongest inhibitions of the four pathobionts together with AMBR074’s- and AMBR024’s CFS, but only for *P. intermedia*, *P. gingivalis* and *F. nucleatum*.

### Probiotic incorporation in biofilms

Since bacteria in the oral cavity are only temporarily in a planktonic state, the colonisation of probiotic candidates into the biofilms was also evaluated using strain-specific primers (Table 2) or species-specific primers (for *S. salivarius* spp.; Supplementary Table 1).

**Table 2:** Sequences of primers for probiotic detection (5’-3’).

Bacterial species	Forward primer	Reverse primer
*Lactiplantibacillus pentosus* KCA1	AATCCACTCGTGATTCCCATAC	TCAAGCTCGCAAAGGTAGTC
*Lacticaseibacillus rhamnosus* GG	CGTAGCTCTTTGCGTCATCT	CGCATTGTATGCAGCCTTATTC
*Lactiplantibacillus plantarum* WCFS1	GCCACAACACTTCAGCAATAC	GTGCCATACACCCTGGTAAG
*Limosilactibacillus fermentum* AMBV001	GGCTCAACTTGCACCTATCA	TGATTCATCACGATGGTGTTCT
*Limosilactibacillus reuteri* AMBV038	GGTAACTGATTTGACAGCACAAG	GCCGGTTGGTCATCCTTATTA
*Limosilactibacillus reuteri* AMBF471	CGTGAGATTCTTGACGCCATAA	CCGCTGAATATCTTGGACAACT
*Streptococcus salivarius* AMBR024	ATGCGATTCCTGCTCTACATAC	TCCCTGCTCCTCCTTGAATA
*Streptococcus salivarius* AMBR074	GGCTGGCTTAACCTACTCA	CTCCTCAGCGGTCTCAAA
*Streptococcus salivarius* AMBR075	CGGAGGTGCTATGGCTAAATAC	CCTATTATGGTATGGCCGCTTT
*Streptococcus salivarius* AMBR158	GTCAATAGTGTAGTTGAGGAACTGA	CTGGCTCTTTCTCTTGCTTCT

**Fig. 2:**
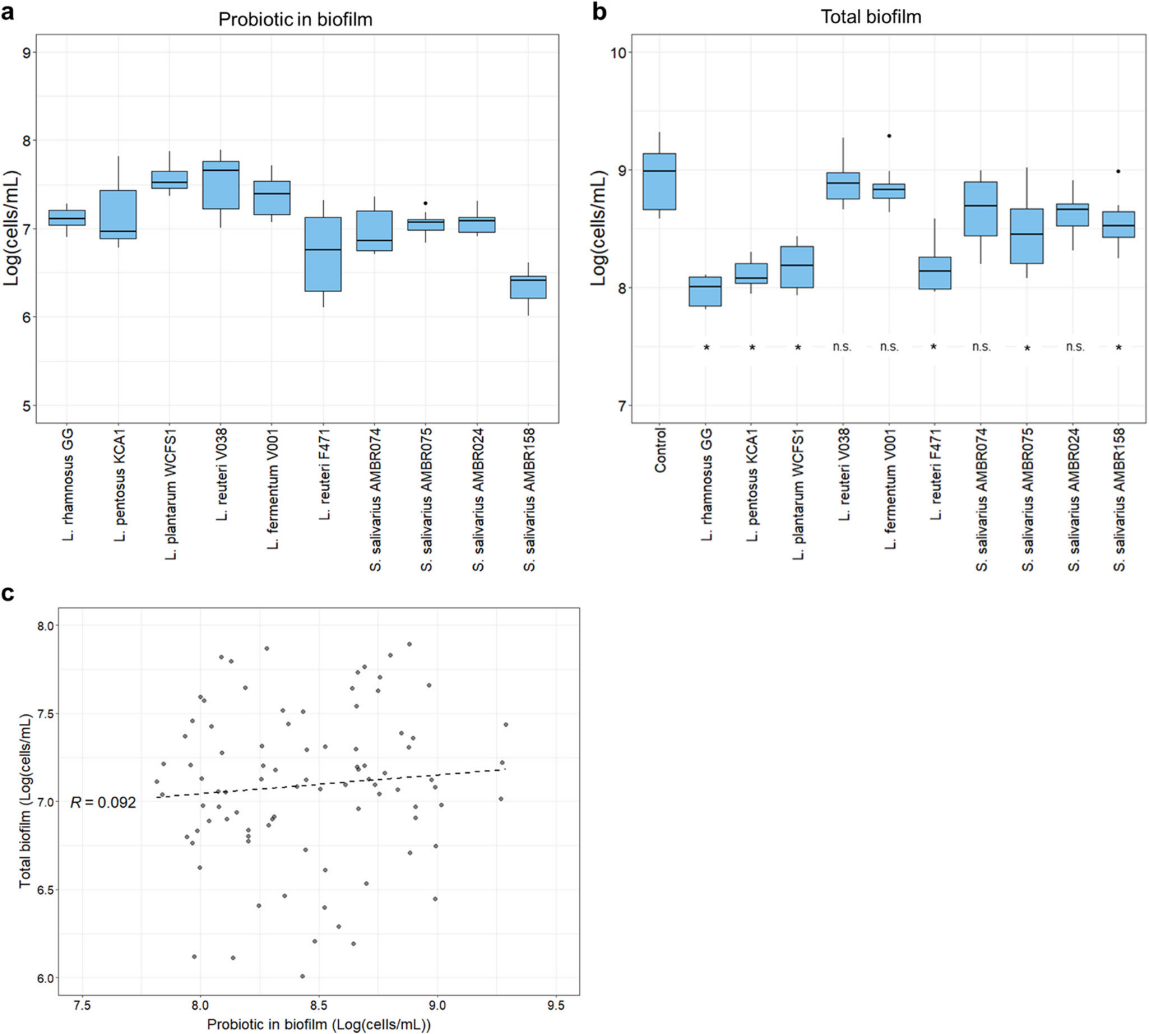
Probiotic prevalence does not affect total biofilm counts. Probiotic prevalence in biofilms (**a**), total cells in the biofilms (**b**) and both plotted against each other (**c**). Probiotic abundance and total biofilm are presented as a boxplot of the log_10_ cells per milliliter (log(cells/mL)) recovered from the discs of each probiotic per experiment (Tukey style boxplot with median centre line, IQR at 1.5, upper and lower hinges at 25th and 75th percentiles and whiskers at 1.5 IQR from the hinges). No correlation between the prevalence of the probiotic and the total biofilm was observed. Statistical differences in probiotic prevalence between conditions can be found in Supplementary Table 2. For the total biofilms, statistical differences from the control are depicted by an asterisk and otherwise as n.s. (*n* = 9; nonparametric Kruskal-Wallis with Dunn’s test).

WCFS1, V038 and V001 were the most abundant of the candidates in the multispecies biofilms, averaging 3.14*10^7^ cells/mL (Fig. 2a). LGG, KCA1, AMBR074, AMBR075 and AMBR024 showed similar colonization of 10% of the added concentration (1.18*10^7^ cells/mL), while F471 and AMBR158 colonized around an average of 3.43*10^6^ cells/mL.

### Inhibition of pathobionts in biofilms

**Fig. 3:**
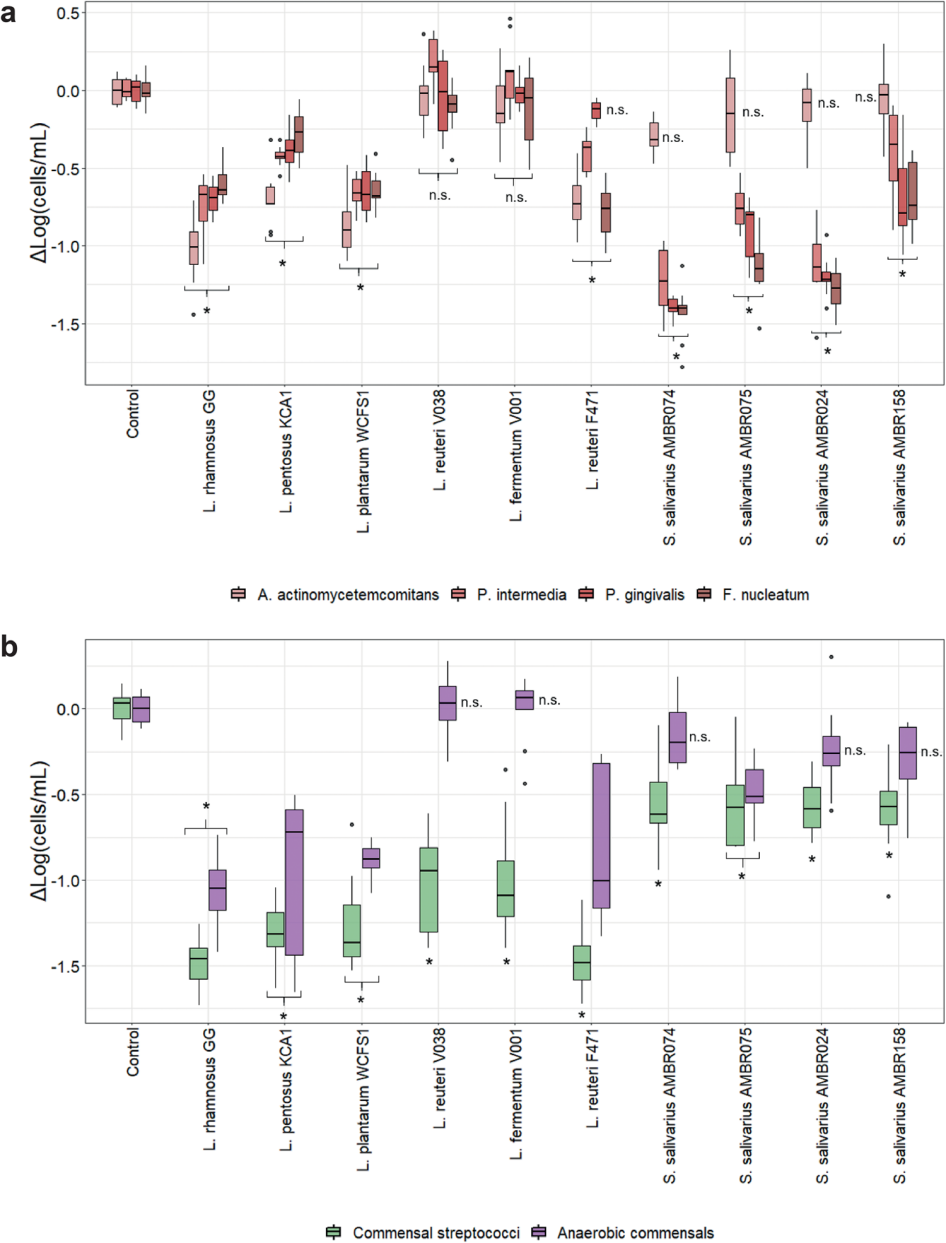
Probiotic effects on oral bacteria in biofilms. Changes in prevalence of (**a**) pathobionts and (**b**) commensals in multispecies biofilms caused by the addition of the probiotic candidates during biofilm formation. Relative changes in abundance are presented as a boxplot of the change in log_10_ cells per milliliter (log(cells/mL)) from the control (Tukey style boxplot with median centre line, IQR at 1.5, upper and lower hinges at 25th and 75th percentiles and whiskers at 1.5 IQR from the hinges). n.s.: nonsignificant from control biofilm. Statistically significant differences from the control biofilm are depicted as an asterisk (*n* = 9, ANOVA with Tukey HSD).

As a primary goal, the relative decrease of pathobionts in the biofilms was evaluated (Fig. 3). While *A. actinomycetemcomitans’* prevalence in one of the control biofilms differed significantly from the other two independent controls, the log change caused by the candidates remained similar. *A. actinomycetemcomitans* was significantly reduced by LGG, WCFS1, KCA1 and F471 by 0.7–1.4 log. V038, V001 and the *S. salivarius* strains did not significantly affect *A. actinomycetemcomitans*.

AMBR074 and AMBR024 showed a reduced prevalence of *P. intermedia*, *P. gingivalis* and *F. nucleatum* of around 1–1.5 log. AMBR075 shared this effect on *P. gingivalis* and *F. nucleatum*, but a reduced effect on *P. intermedia*. AMBR158 affected these three pathobionts, but to variable degrees, possibly due to its lower incorporation in the biofilms (Fig. 2). However, the antimicrobial effects of the other probiotics did not positively correlate with their biofilm prevalence. While LGG, KCA1 and WCFS1 also affected *P. intermedia*, *P. gingivalis* and *F. nucleatum*, their extent was lower than the *S. salivarius* strains and averaged at 1 log lower decrease (Fig. 3). F471 shared these observations for *P. intermedia* and *F. nucleatum*, but not for *P. gingivalis*. V001 and V038 did not reduce the prevalence of the pathobionts and slightly increased the prevalence of *P. intermedia*.

**Fig. 4:**
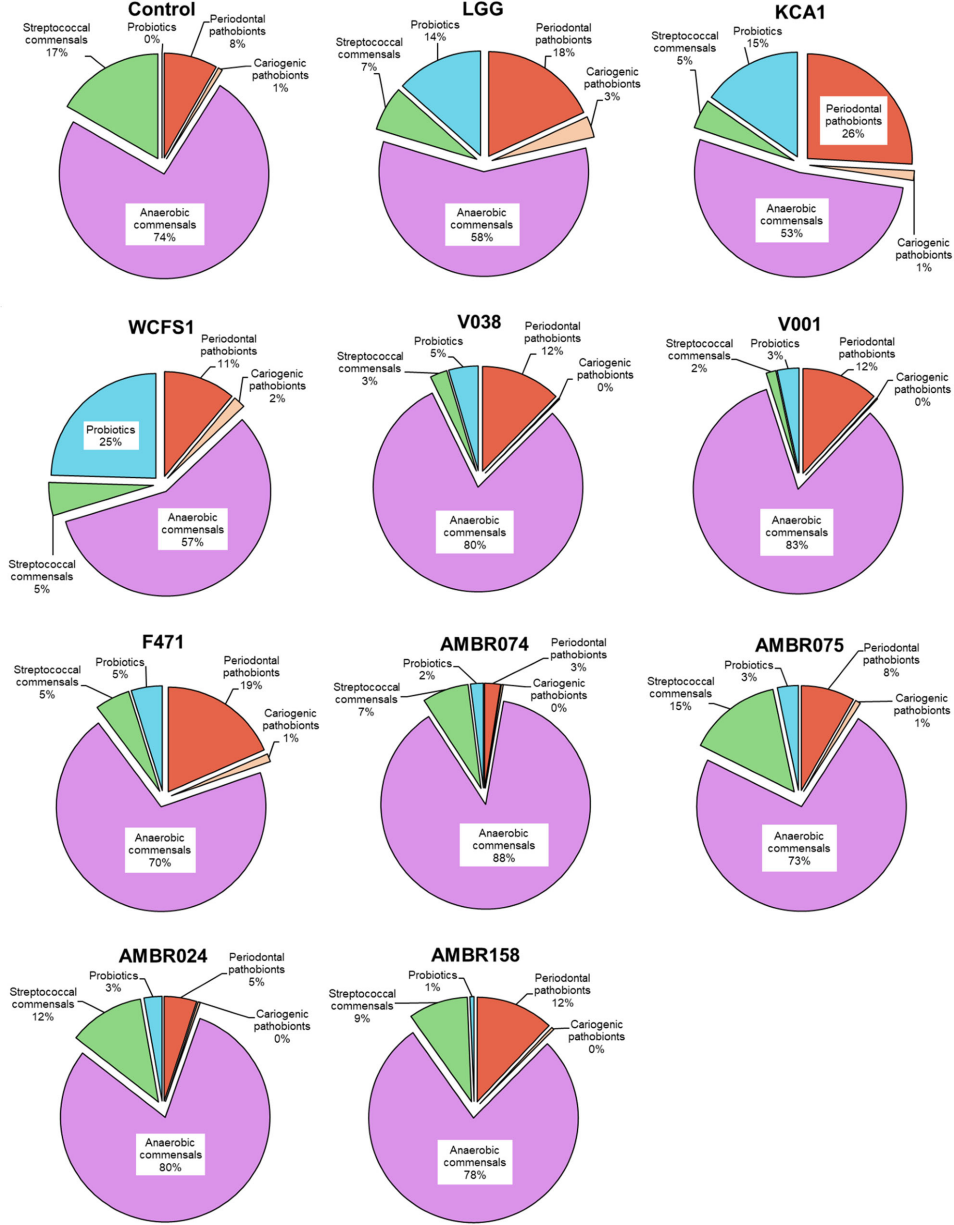
Probiotic effects on the proportional composition of the biofilms. Combined data of Figs. 1 & 2 presented as pie charts of the percentage composition of these biofilms divided into probiotics, periodontal pathobionts, cariogenic pathobionts, anaerobic commensals and streptococcal commensals.

Cariogenic streptococci were incorporated into this model because they can be present in the subgingival biofilm and because it has been demonstrated that a reduction in periopathobionts can shift the ecology toward a more cariogenic microbiota^19^. This shift was not observed in the experiments when any of the candidate probiotics were administered (Fig. 4).

### Inhibition of commensals in biofilms

While the decrease in pathobiont prevalence is often the goal, antimicrobial mechanisms might also affect commensal species. All probiotics showed antagonism towards the commensal streptococci (Fig. 3). Especially the lactobacilli, including the pathobiont ineffective V038 and V001, showed significant decreases in commensal streptococci. While the *S. salivarius* strains also affected the commensal streptococci, these decreases were less pronounced. The most abundant commensal, *V. parvula*, was also significantly affected by the lactobacilli effective against the periodontal pathobionts. V038 and V001 did not affect this commensal and the *S. salivarius* strains only caused a low, mostly non-significant decrease in *V. parvula*.

### Biofilm ecology

The combined effects of the probiotic on both pathobionts and commensals resulted in total biofilm formation (Fig. 2b) as well as compositional changes herein (Fig. 4). Total biofilm formation was lowered by 1 log by LGG, KCA1, WCFS1 and F471 due to their effects on the pathobionts as well as the decreases in the abundant commensal streptococci and *V. parvula*. These changes resulted in proportional decreases in the anaerobic commensals (down to 53–58%) and commensal streptococci (down to 3–5%), resulting in the probiotic being 14–25% of the composition (for LGG, KCA1 and WCFS1) but also proportionally increasing the periodontal pathobionts up to 18–25% (for LGG, KCA1 and F471).

While not affecting the other oral bacteria, V038 and V001 did affect the commensal (and cariogenic) streptococci, resulting in an equivalent proportional increase in the other species.

The effects of AMBR074, AMBR024 and to a lesser extent AMBR158 on the periodontal pathobionts together with more limited effects on the commensals, resulted in only proportional decreases in the pathobionts (down to 5-3% for AMBR024 and AMBR074) with little changes in the total biofilm numbers and other species.

## Discussion

While the evidence for the use of probiotics to maintain or restore oral health is increasing, due to the variety of microbiological causes that result in a similar pathology, not just any probiotic strain will function to resolve the diverse underlying causes of similarly presenting pathologies. The increasing understanding that periodontitis is caused by a ‘personalized pathology'^20^, differing from patient to patient, may move the field of oral probiotics towards more ‘precision probiotics'^21^, selecting the correct probiotic on a case-per-case basis with increased efficiency.

Some of the *Lactobacillus* strains used in the current study (LGG, KCA1 and WCFS1) are known to possess both antimicrobial and immunomodulating properties, leading to the alleviation of other inflammatory diseases^22–24^. These properties might make these translationally useful in the management of periodontitis.

A selection of novel bacteria was made based on unique strains available in the Lab of Applied Microbiology and Biotechnology (AMB) from previous research isolated from different sources: human niches (upper respiratory tract, vagina) and fermentation sources.

A thorough screening approach was applied based on the rationale that the strains had to be safe, be applicable (being robust and showing lifestyle flexibility, as described for lactobacilli by Duar et al.^25^, and have the capacity to exert beneficial functions in the oral cavity, including microbiome modulation, immune modulation, and epithelial barrier enhancement. Key properties were substantiated with laboratory tests, genome screening, and information available in literature^22^.

The strains were evaluated on single species inhibition of four periodontal pathobionts with a classical agar-based technique and a novel flow cytometric (FCM) approach using the cell-free supernatant of the probiotic. While the inhibition of *P. intermedia, P. gingivalis* and *F. nucleatum* were similar between biofilms and FCM, a notable difference was that in the agar setup, *A. actinomycetemcomitans* was inhibited by the *S. salivarius* strains while this was not the case in the FCM assay. This lack of inhibition was also present in the biofilms. On the other hand, four of the lactobacilli were able to consistently inhibit *A. actinomycetemcomitans* in all three setups.

While many of the probiotics were able to incorporate into the biofilms, their degree of colonization varied between species, though this did not seem to correlate with their antimicrobial potential. This combined with their different antimicrobial effects on both pathobionts and commensal species resulted in compositional changes that can be grouped into three categories: ineffective (V001, V038), *Lactobacillus* type (broad spectrum antimicrobial against most oral species; LGG & WCFS1, followed by KCA1 and F471) and *S. salivarius* type (only effective against *P. intermedia*, *P. gingivalis* and *F. nucleatum*; AMBR074 & AMBR024 followed by AMBR075 and AMBR158 to a lesser degree). While some of the probiotics’ antimicrobial behaviour could be loosly grouped according to their broad genera, the potency of their antimicrobial potential differed significantly between strains.

While agar-based techniques have long been the gold standard for evaluating antimicrobial potential, the biological relevance of agar growth as well as the relatively low throughput and somewhat qualitative nature of the results might favour the use of more modern techniques with higher throughput and more quantitative results. One such technique used in the current study is the optical single-cell analysis technique, flow cytometry. The ability to analyse thousands of cells in mere seconds with a large collection of dyes and molecular probes allows for a variety of applications^26,27^. FCM has proved to be valuable in screening novel antimicrobials and their effectiveness against pathobionts^28,29^. Especially membrane integrity is easy to assess through the membrane-impermeable propidium iodide, allowing for rapid determination of antimicrobial peptides affecting the membrane permeability of *F. nucleatum* or *S. mutans* for example^30^.

While FCM has been used to accurately count probiotics and evaluate their viability (including nonculturable cells: VBNC)^31^, to our knowledge, FCM has yet to be used to screen the effectiveness of probiotics/postbiotics on pathobionts.

Due to the extra noise that another cell type would give in the flow cytometry of the pathobionts, cell-free supernatant of the probiotic was chosen. Because this CFS lacks living cells, it falls under the newly defined definition of a postbiotic, a “preparation of inanimate microorganisms and/or their components that confers a health benefit on the host“^18^. While these postbiotics have been found to share the naturally secreted antimicrobials with the probiotic^32^, probiotic mechanisms that are actively induced by contact with other bacteria are lost due to the inertness of the postbiotic. The FCM evaluation of the CFS showed remarkable similarities with the biofilms, while the agar setup showed a drastic difference between these two. The agar setup showed that the *S. salivarius* strains were able to inhibit *A. actinomycetemcomitans*. This inhibition was not present in the FCM assay which aligned more with the lack of inhibition in the biofilms. Conversely, LGG and F471 did show inhibition of *P. intermedia* and *F. nucleatum* respectively in the FCM but not the agar assay.

An important discrepancy between the postbiotic FCM and probiotics within the biofilms is the difference in the magnitude of pathobiont inhibition. While the strongest lactobacilli and streptococci candidates showed a very similar inhibition in the FCM assay on *P. intermedia*, *P. gingivalis* and *F. nucleatum*, the decrease of these three pathobionts in the biofilms was significantly stronger by the streptococci. Since the probiotic abundances were close to equal, the difference is likely due to the streptococcal antimicrobial mechanism being more effective within biofilms or the presence of other biofilm-related antimicrobial mechanisms (e.g. only expressed in biofilms or anti-biofilm mechanisms).

These differences in the strain-specific effectiveness may be due to the targets of their produced antimicrobials. Lactobacilli are well-known for their production of bacteriocins^33,34^, however, most of these peptides are either target cell wall integrity or synthesis^33,35^, making the outer membrane of the Gram-negative periodontal pathobionts an innate resistance mechanism. Despite this being the general consensus, some bacteriocins seem to be able to also affect the membrane. As an example, the well-known ‘Nisin’ bacteriocin is known to affect cell wall synthesis^33^, as well as form membrane pores, resulting in the killing of Gram-negatives such as *E. coli*^36^. Nisin has also been found to be effective against *A. actinomycetemcomitans*, *P. intermedia*, *P. gingivalis*, *F. nucleatum*, and *Treponema denticola*^37^. Other bacteriocin-like peptides have also been found to be effective against periodontal pathobionts^38,39^.

Even though lactobacilli are usually the focus of probiotic use due to their long history of safe use and extensive research, lactobacilli are not the most prevalent in healthy dental biofilms. Since *Streptococcus* species are more native to and abundant in the oral cavity, they might be more effective since they are inherently already adapted to the niche. Strains such as *S. dentisani* and *S. salivarius* are increasingly being used for oral health^40–42^. In line with these findings, the *S. salivarius* AMBR074 and AMBR024 in the current study showed the largest decreases of the periodontal pathobionts (except for *A. actinomycetemcomitans*), making them promising candidates.

As for the differences in effectiveness, while *S. salivarius* strains are known to produce bacteriocins such as Salivaricin B, it does not show antimicrobial activity against Gram-negative bacteria^41^. This might explain the effect of *S. salivarius* strains on commensal streptococci. However, *S. salivarius* strains, like several other streptococci, are able to produce another important biofilm-shaping antimicrobial, hydrogen peroxide (H_2_O_2_)^43,44^. The observation that *A. actinomycetemcomitans*, which unlike most periodontal pathobionts is naturally resistant to H_2_O_2_^45^, was not as affected by the *S. salivarius* strains supports the probability that H_2_O_2_ is likely their most effective antimicrobial in these biofilms.

We hypothesised that a probable determining factor of probiotic effectiveness was the prevalence of the probiotic in the biofilms. While more incorporation might lend to greater opportunity to inhibit other species, this was not the case as the biofilm-abundant V038 and V001 did not show more antimicrobial effects than the significantly less abundant LGG, for example. While AMBR158 showed the lowest abundance of the *S. salivarius* strains as well as the weakest antimicrobial activity, for all the other candidates, the abundance of the probiotic candidate did not correlate with their antimicrobial effects.

While a few commensal streptococci have been implicated in oral disease (e.g. *S. gordonii* in periodontitis^46^), some display clear antagonism towards periodontal pathobionts, are abundantly present in healthy individuals and can play an important role in preventing dysbiosis^43,47^.

While V038 and V001 did not show any antimicrobial effectivity against the periodontal pathobionts, they, as well as the other lactobacilli, showed antimicrobial effects against the oral streptococcal species. *L. rhamnosus* GG has been long known for its anti-caries activity^48,49^. These antimicrobials are presumably also effective against other *Streptococcus* species.

In addition to this, LGG, WCFS1, KCA1 and F471 also showed significant decreases in *V. parvula*, which in this model is considered as an anaerobic commensal, a non-pathogenic species. However, recent research suggests that *Veillonella* species might have a role as an accessory pathogen^50,51^, making reducing these potentially interesting. Contrary to this inhibition, several *Streptococcus* species including *S. salivarius* have been found to interact with *Veillonella* species in biofilm formation^52^, which might explain the little antagonism between these species in the current study.

Based on this all, combining different probiotics might result in a more diverse and concerted antimicrobial activity^53^. Combining LGG or WCFS1 with AMBR074 or AMBR024 might result in a synergistic effect on all periodontal pathobionts as well as *V. parvula*, however, recent research also shows that these antimicrobials could act antagonistically towards each other instead of synergistically^54^. Additionally, effects on commensal streptococci as well as each other’s probiotic incorporation are also to be expected.

While the used biofilm model allows for a comparative evaluation of the probiotic candidates, while mimicking in vivo oral biofilms, it lacks several important driving pathobionts of periodontitis as well as important commensals with potentially yet unknown influences on the microbial community. How these are affected by the probiotic candidates cannot be estimated by this model. More expansive models or more patient/pathology-specific models can provide valuable insights into how probiotics can influence the oral microbiome. While establishing models with hundreds of species might be impossible, including more prominent bacterial genera remains desirable. Alternatively, in vivo validation can subvert this limitation, but this has important limitations in sample size and variations in host microbiomes.

Other important probiotic characteristics such as in vivo oral colonization and host inflammatory modulation of the candidates were not evaluated in this study. The simultaneous colonization of a clean surface by both the probiotic and oral community was evaluated, while in vivo presence of already formed biofilm seems to limit the colonization of probiotics^55^. Additionally, the model does not account for oral dynamics such as friction, salivary flow, and oral hygiene practices, which may also limit the colonization of probiotics.

Most of the selected strains used in this study were from a non-oral niche, which might hinder their ability to colonize the oral microbiome. However, other members of their species can occur endogenously in the oral microbiome or have shown their potential as probiotics in clinical trials. If these (muco-)adhesive mechanisms are conserved within their species, this will likely allow the candidate strains to also adhere to the oral mucosae and dental pellicle. In a recent study on KCA1, LGG and WCFS1, a novel microcapsule formulation was used which allowed them to colonise the skin microbiome (to which they were not native) and suppress the local pathobionts^22^. With the proper delivery method, these non-oral strains are expected to at least adhere to the oral tissues.

Whether the investigated strains will permanently colonize the oral microbiome and show the same antagonism towards periodontal pathobionts requires further investigation with clinical trials.

In conclusion, the evaluated probiotic candidates showed species-specific differences in their antimicrobial potential, targets (pathobionts and commensals) as well as in vitro biofilm incorporation. Biofilm incorporation was not a good predictor of the magnitude of their effect. Postbiotic effects on pathobionts analysed with FCM showed predictive antimicrobial effectiveness of the probiotics’ effects in biofilms, but not the magnitude of antimicrobial effects. Further investigation using in vivo trials will determine if these strains can indeed function as oral probiotics.

## Methods

### Bacteria and media

*Lactobacillus* probiotic candidates were cultured anaerobically on Man, Rogosa and Sharpe agar (MRS; Merck, Darmstadt, Germany) at 37 °C (Table 3). Streptococcal probiotics and all oral bacteria were cultured on blood agar as described previously^56^ (Table 3 & 4).

**Table 3:** Probiotic candidates.

Name	Short	Niche	Reference
*Lacticaseibacillus rhamnosus* GG	LGG	Gastrointestinal tract	Kankainen et al.^74^
*Lactiplantibacillus pentosus* KCA1	KCA1	Vagina	Anukam et al.^75^
*Lactiplantibacillus plantarum* WCFS1	WCFS1	Saliva	Kleerebezem et al.^76^
*Limosilactobacillus reuteri* AMBV038	V038	Vagina	Lab of Applied Microbiology and Biotechnology (LAMB)
*Limosilactobacillus fermentum* AMBV001	V001	Vagina	
*Limosilactobacillus reuteri* AMBF471	F471	Fermentation	
*Streptococcus salivarius* AMBR074	R074	Respiratory tract	
*Streptococcus salivarius* AMBR075	R075		
*Streptococcus salivarius* AMBR024	R024		
*Streptococcus salivarius* AMBR158	R158

**Table 4:** Oral bacteria in the biofilm model.

Name	Class	Reference
*Aggregatibacter actinomycetemcomitans* ATCC 43718	Periodontal pathobiont	Slomka et al.^56^
*Prevotella intermedia* ATCC 25611		
*Porphyromonas gingivalis* ATCC 33277		
*Fusobacterium nucleatum* ATCC 20482		
*Actinomyces viscosus* DSM 43327	Anaerobic commensal	
*Actinomyces naeslundii* ATCC 51655		
*Veillonella parvula* DSM 2008		
*Streptococcus mutans* ATCC 20523	Cariogenic streptococci	
*Streptococcus sobrinus* ATCC 20742		
*Streptococcus oralis* DSM 20627	Commensal streptococci	
*Streptococcus sanguinis* LM14657		
*Streptococcus gordonii* ATCC 49818		
*Streptococcus mitis* DSM 12643		

Agar inhibition assays were performed on BHI-2-gluc agar (modified brain heart infusion (BHI) broth according to Àlvarez et al. (2013)^57^ supplemented with 0.5 % (w/v) D-glucose and 1% (w/v) agar). Probiotics were grown in BHI-2-gluc without mucine before FCM assays and biofilm assays.

### Selection of candidates

A selection of different bacteria was made based on unique strains from previous research isolated from different sources: human niches (saliva, gastrointestinal tract, upper respiratory tract, vagina) and fermentation. A thorough screening approach was applied based on the rationale that the strains had to be safe, be applicable (being robust and showing lifestyle flexibility, as described for lactobacilli by Duar et al.^25^), and have the capacity to exert beneficial functions in the oral cavity, including microbiome modulation, immune modulation, and epithelial barrier enhancement (the latter two not evaluated in the current study). Key properties were substantiated with laboratory tests, genome screening, and information available in literature^22^.

### Antagonistic agar assay

Probiotic overnight cultures were adjusted to approx. 10^8^ cells/mL and 7 µL were spotted on a BHI-2-gluc agar plate. After 24 h of anaerobic growth at 37 °C, the pathobionts were spotted adjacent to the probiotic and incubated for another 24–48 h until the growth of the control spot was visible. A calibrated photo was taken of the plates and the reduction in diameter compared to the control was measured in ImageJ (Supplementary Fig. 1).

### Flow cytometry (FCM) assay

Probiotic candidates and pathobionts were cultured overnight in BHI-2-gluc without mucin. Afterwards, cells were centrifuged (5000 x g for 5 min) and the supernatant was filter sterilized through a 0.22 µM membrane filter to obtain the cell free supernatant (CFS, pH ranges naturally between 6–6.4). Spent pathobiont medium (to keep pathobionts in stationary phase) was also filter sterilized and pH adjusted to 6–6.4 with 0.1 M HCl.

Pathobionts were cultured overnight in BHI and adjusted to 10^8^ cells/mL (evaluated with FCM) before centrifugation and resuspension in probiotic CFS or spent pathobiont medium. After anaerobic incubation at 37 °C for 6 h, samples were taken for FCM evaluation.

Samples were diluted with phosphate-buffered saline (PBS) to 10^5^ to 10^6^ cells/mL and stained with SYBR™ Green I (SG; 1x final; Invitrogen, Thermo Fisher Scientific, Waltham, Massachusetts, USA) and propidium iodide (PI; 4 µM final Thermo Fisher Scientific, Waltham, Massachusetts, USA) and incubated for 20 min at 37 °C. Flow cytometry (FCM) analysis was performed using a FACSVerse cytometer with a blue 488-nm laser and a red 640-nm laser (BD Biosciences, Belgium). Forward scatter (FSC-A), side scatter (SSC-A), green- (FITC-A) and red fluorescence signals (PerCP-CY5.5-A) were used to characterize and gate live/intact cells and dead/damaged cells (uptake of propidium iodide due to membrane damage: increasing red fluorescence with decreasing green fluorescence (FITC-A) through fluorescence resonance energy transfer (FRET); Supplementary Figures 1 & 2).

### Primer design probiotics

Unique strain-specific genes were selected by comparing all present (hypothetical and functional) genes from whole genome sequences present in NCBI (Eilers, Delanghe et al. in preparation). This gene library was compared to all genomes of its species, rare genes (only present in up to 10 genomes) were selected and compared in blast to check whether their sequences were unique compared to all sequenced samples. Only unique sequences were further tested and used to develop primer sequences for qPCR with intercalating dyes, using the PrimerQuest™ Tool (Integrated DNA Technologies). Standard curves were used to estimate bacterial DNA concentrations in the samples and derived from serially diluted DNA from an overnight culture. The selected primer pair was validated by testing cross-reactivity for different strains from *Lactobacillus* sp., *Streptococcus* sp. and *Staphylococcus* sp. and oral bacteria resulting in no amplified sequences except for the intended strain (Eilers, Delanghe et al. in preparation). Used primers are presented in Table 2.

### Biofilm model

To evaluate the probiotic incorporation and effect on oral bacteria, the previously described multispecies oral biofilm model by Slomka and colleagues (2018)^56^ without *S. salivarius* TOVE-R was used.

Briefly, a multispecies community of 13 oral bacteria was established in a bioreactor and after stabilization, a biofilm seed culture was sampled. Probiotic candidates were grown overnight in BHI-2-gluc and adjusted to 1 × 10^8^ cells/mL (evaluated with FCM) and were added to a 1:10 dilution of the bioreactor seed culture (also 1 × 10^8^ cells/mL). Biofilms were formed on calcium-deficient hydroxyapatite discs (8 mm diameter) for 24 h micro-aerophilic (6% oxygen) at 37 °C before cells were recovered from the biofilms (biofilm disruption done enzymatically with trypsin and mechanically by vortexing and low-frequency sonification to a final volume of 0.5 mL). 90 µL of this was viability treated with PMAxx^58^ (Biotium, Hayward, CA, USA) and extracted with the QIAamp® DNA Mini Kit (QIAGEN, Hilden, Germany) to a final volume of 200 µL per sample. Taking dilutions into account, DNA was analysed through qPCR according to Herrero et al.^59^, with the use of revised sequences of the primers for amplicons to above 150 base pairs^58^. Primer and probe sequences and qPCR assay conditions are listed in Supplementary Table 1.

#### About the biofilm model

Unlike classical microbial disease aetiology where the presence of a single pathogen is cause of the disease, all species used in this model are naturally occurring symbionts of the oral cavity. Some of these species have been found to play important roles in the development and progression of oral disease under certain conditions. However, their presence alone does not necessarily imply a diseased state. Therefore, these species are commonly referred to as ‘pathobionts’, a symbiont that is otherwise harmless but can cause a pathology when certain conditions are met^60^. While in some cases the line between beneficial and harmful can be clearly drawn, the messy nature of biology tends to prefer blurred lines. A relevant example in the ‘probiotic context’ of this paper is a report on the commonly used probiotic *Lacticaseibacillus rhamnosus* being the cause of sepsis in a cardiosurgical patient^60,61^. Harm or benefit is defined by the context.

In the context of this model, the disease-driving properties of *A. actinomycetemcomitans*, *P. gingivalis* and *P. intermedia* in the development and progression of periodontitis, and the cariogenic properties of *S. mutans* and *S. sobrinus*, are well characterized in literature^17,62–66^. The line becomes blurrier with *F. nucleatum*. While still deemed a pathobiont due to its indirect, but central role as a bridge-organism in periodontitis^67^, its direct role in disease progression is more nuanced^68^. For their causal roles, these symbionts were grouped as **‘periodontal pathobionts’** or **‘cariogenic pathobionts’**.

The six commensal species used in this model have been found to be present (and abundant) in both health-associated and disease-associated biofilms, with suspected roles in both. Nevertheless, for simplicity and clarity, a classification into two groups was made in this model, based on their antagonism towards periodontal pathobionts.

Lacking clear antagonism to periodontal pathobionts, the *Actinomyces* spp. and *V. parvula* were grouped as **‘anaerobic commensals’** in this model. The ubiquitous *Actinomyces* species are found in oral health, e.g. playing an important role in maintain pH homeostasis, preventing the outgrowth of cariogenic pathobionts. However *Actinomyces* species are frequently found in increased abundances in periodontitis^69^. Whether *Actinomyces* species are actively involved in periodontitis is still unclear, making them unlikely to be drivers of periodontitis. *V. parvula* is also frequently found to be present in and aid formation of both health- and disease-associated biofilms^67,70^. Unlike the *Actinomyces* species, evidence is emerging that *V. parvula* is actively involved in stimulating the virulence of *P. gingivalis*^51^.

As one of the most abundant constituents of the oral microbiome, the second group of commensals in this model consists of the ‘**commensal streptococci**’. These are also naturally present in a health-associated microbiome, but are also required for disease initiation. As primary colonizers they are required for the adhesion and biofilm formation of the often pathogenic secondary colonizers^67,71^, thus also having an indirect role in periodontitis development. Especially *S. gordonii* has been well characterized to aid *P. gingivalis* incorporation into biofilms^72^, but was also found by the same group to mitigate the harm caused by *P. gingivalis*^73^. Additionally, several streptococcal species also show clear antagonism towards periodontal pathobionts through the production of hydrogen peroxide^43^. While also involved in periodontitis, these commensal streptococci are known to be important in maintaining the microbiome-host homeostasis, preventing dysbiosis^66^.

### Statistics

Statistical analysis was performed in R 4.0.0 (https://cran.r-project.org/). Parametric data were analysed with an ANOVA with Tukey HSD multiple comparisons (95% CI) performed to evaluate statistical differences. Nonparametric data were analysed with a Kruskal-Wallis with Dunn’s test (for probiotic prevalence in the biofilms and total biofilms). Differences in FCM data were evaluated only on the live cells.

### Reporting summary

Further information on research design is available in the Nature Research Reporting Summary linked to this article.

## Supplementary information

Revised supplementary information

Reporting Summary

## Data Availability

The data that support the findings of this study are available from the corresponding author upon reasonable request.
